# Pathogenic Roles of Autoantibodies and Aberrant Epigenetic Regulation of Immune and Connective Tissue Cells in the Tissue Fibrosis of Patients with Systemic Sclerosis

**DOI:** 10.3390/ijms21093069

**Published:** 2020-04-27

**Authors:** Chang-Youh Tsai, Song-Chou Hsieh, Tsai-Hung Wu, Ko-Jen Li, Chieh-Yu Shen, Hsien-Tzung Liao, Cheng-Han Wu, Yu-Min Kuo, Cheng-Shiun Lu, Chia-Li Yu

**Affiliations:** 1 Division of Allergy, Immunology & Rheumatology, Taipei Veterans General Hospital & National Yang-Ming University, #201 Sec. 2, Shih-Pai Road, Taipei 11217, Taiwan; darryliao@yahoo.com.tw; 2 Department of Internal Medicine, National Taiwan University Hospital and National Taiwan University College of Medicine, #7 Chung-Shan South Road, Taipei 10002, Taiwan; hsiehsc@ntu.edu.tw (S.-C.H.); dtmed170@yahoo.com.tw (K.-J.L.); tsichhl@gmail.com (C.-Y.S.); chenghanwu@ntu.edu.tw (C.-H.W.); 543goole@gmail.com (Y.-M.K.); b89401085@ntu.edu.tw (C.-S.L.); 3 Division of Nephrology, Taipei Veterans General Hospital & National Yang-Ming University, #201 Sec. 2, Shih-Pai Road, Taipei 11217, Taiwan; thwu@vghtpe.gov.tw; 4 Institute of Clinical Medicine, National Taiwan University College of Medicine, #7 Chung-Shan South Road, Taipei 10002, Taiwan

**Keywords:** non-coding RNA, microRNA, long non-coding RNA, Wnt/catenin signal pathway, tissue fibrosis, myofibroblast trans-differentiation, pro-fibrogenic cytokines, TGF-β, systemic sclerosis

## Abstract

Systemic sclerosis (SSc) is a multi-system autoimmune disease with tissue fibrosis prominent in the skin and lung. In this review, we briefly describe the autoimmune features (mainly autoantibody production and cytokine profiles) and the potential pathogenic contributors including genetic/epigenetic predisposition, and environmental factors. We look in detail at the cellular and molecular bases underlying tissue-fibrosis which include trans-differentiation of fibroblasts (FBs) to myofibroblasts (MFBs). We also state comprehensively the pro-inflammatory and pro-fibrotic cytokines relevant to MFB trans-differentiation, vasculopathy-associated autoantibodies, and fibrosis-regulating microRNAs in SSc. It is conceivable that tissue fibrosis is mainly mediated by an excessive production of TGF-β, the master regulator, from the skewed Th2 cells, macrophages, fibroblasts, myofibroblasts, and keratinocytes. After binding with TGF-β receptors on MFB, the downstream Wnt/β-catenin triggers canonical Smad 2/3 and non-canonical Smad 4 signaling pathways to transcribe collagen genes. Subsequently, excessive collagen fiber synthesis and accumulation as well as tissue fibrosis ensue. In the later part of this review, we discuss limited data relevant to the role of long non-coding RNAs (lncRNAs) in tissue-fibrosis in SSc. It is expected that these lncRNAs may become the useful biomarkers and therapeutic targets for SSc in the future. The prospective investigations in the development of novel epigenetic modifiers are also suggested.

## 1. Introduction

**Figure 1:**
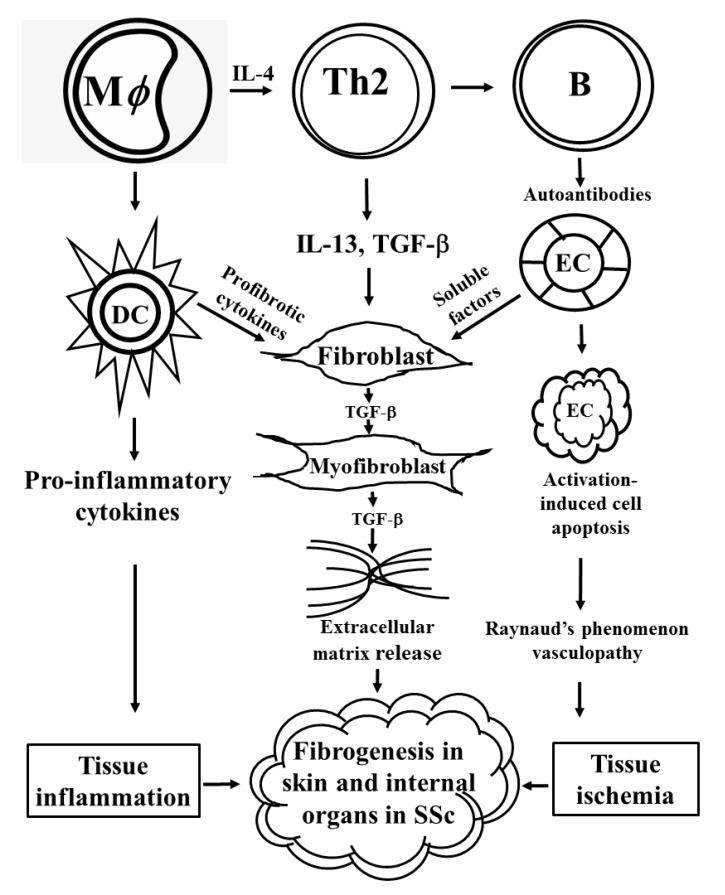
Autoimmune-mediated fibroblast-to-myofibroblast trans-differentiation, vascular endothelial cell (EC) damage, tissue ischemia, tissue inflammation, and finally tissue fibrosis in patients with systemic sclerosis.

Systemic sclerosis (SSc) is a systemic autoimmune disease characterized by the presence of a broad spectrum of autoantibodies, vascular endothelial damage, non-infective inflammation, and tissue fibrosis in the skin and internal organs, especially the lungs [1,2,3,4,5,6,7,8]. Clinically, endothelial dysfunction presenting as Raynaud’s phenomenon is a starting manifestation in patients with SSc, which is originated from damage to capillary lumens. Subsequently, tissue fibrosis ensues in the skin of hands and extends to internal organs [3,9,10,11,12]. Presumably, both innate [7] and adaptive immune cells [1,2,3,4,8] participate in the eccentric immune responses that skew naïve T cells toward Th2 responses in SSc patients. High levels of Th2 cytokines such as IL-13 and TGF-β have been found in the tissues of SSc patients [3,4,9,10,11,12]. Moreover, these polarized Th2 cells are found adjacent to the fibroblasts (FBs) in connective tissues, causing them to trans-differentiate to myofibroblasts (MFBs). The MFBs are major cells to produce extra-cellular matrix including collagen fibers and fibronectins [4]. In the meantime, B lymphocytes are activated by Th2-derived cytokines, IL-4 and IL-5, as well as macrophage-derived IL-6 to produce diverse autoantibodies that result in vascular endothelial cell damage, tissue ischemia, and chronic inflammation, and eventually tissue fibrosis [8]. Manetti et al. [13] have reported an increase in phenotypic CD3^+^CD31^+^CXCR4^+^ angiogenic T cells (T_ang_) in the peripheral blood and skin tissues of SSc patients with digital ulcers. The increase in this particular T_ang_ phenotype in patients with SSc may reflect an ineffective compensation for angiogenesis and diminished replenishment of CD34^+^CD133^+^VEGFR-2^+^ endothelial progenitor cells in patients with SSc. Trucketet et al. [14] have demonstrated that activated platelets can stimulate endothelial cells and dermal FBs to produce a pro-fibrotic mediator, thymic stromal lymphopoietin (TSLP), in an IL-1β dependent manner in patients with SSc. Benyamine et al. [15] have found that SSc-derived natural killer (NK) cells with a particular phenotype of low expression of CXCR4, NKG2D, and CD69 are the potent inducer of endothelial microparticle release by the activated endothelial cells. On the other hand, the classic innate immune cells such as monocytes and dendritic cells can potently secrete both pro-fibrogenic and pro-inflammatory cytokines to induce tissue inflammation and fibrosis. A scheme demonstrating autoimmune-mediated vasculopathy, tissue inflammation, tissue ischemia, and finally tissue fibrosis in SSc patients is depicted in Figure 1. The autoantibody profiles relevant to respective clinical manifestations and pathological processes in SSc patients are listed in Table 1 [16,17,18,19,20,21,22,23,24,25,26,27,28,29,30,31,32,33,34,35,36,37,38,39,40,41,42,43]. Among these autoantibodies, anti-scleroderma 70 (anti-Scl-70) or anti-double-stranded DNA topoisomerase 1 (anti-TOPO-1), and anti-centromere proteins (anti-CENPs) are the marker autoantibodies of the patients with SSc and its variant, CREST syndrome (acronymed from calcinosis, Raynaud’s phenomenon, esophageal dysmotility, sclerodactidy, and telangiectasis). To clarify the cause-effect relationship of autoantibodies and SSc pathogenesis, Henault et al. [17] have reported that anti-TOPO-1 can directly bind to the surface of FBs. Shen et al. [44] have directly incubated human umbilical vascular endothelial cell line (HUVEC) with the heat-inactivated sera containing anti-CENP-B and anti-TOPO-1 antibodies obtained from SSc patients with Raynaud’s phenomenon and found that the two autoantibodies could induce vascular endothelial cell senescence via a mechanism other than the classic p53-p21 pathway. Nevertheless, the real cause-effect relationship of autoantibodies in the development of specific clinical manifestations particularly the tissue fibrosis in SSc remains to be elucidated.

## 2. The Pathogenic Factors Contributing to the Development of SSc

### 2.1. Genetic Predisposition in Patients with SSc

Recent investigations on SSc have identified more than 30 genetic loci largely belonging to immunity-associated genes including human leukocyte antigen (HLA)-DRB1, DQB1, DQA1, and DPB1, non-HLA (such as STAT4, IRF5, CD247) [45,46,47,48,49] and cancer-associated genes including *Ras*, *Jak/STATs*, *EGFR* [46,50,51]. However, these genetic loci are found only modestly associated in strength with the disease susceptibility. The non-HLA genes associated with SSc are implicated in a wide range of functions including innate and adaptive immune responses, extracellular matrix deposition, cytokine production, and autophagy [42,43,44,45,46,47,48,49]. Thus, these genes are considered related to tissue inflammation, fibrosis, and vasculopathy in patients with SSc [49]. Interestingly, 10% of SSc patients have been observed to produce anti-RNA polymerase III autoantibodies, which are demonstrated significantly relevant to carcinogenesis [52,53,54]. The cancers closely related to these antibodies encompass lungs, breasts, esophagus, urinary bladder, and hematopoietic systems [51]. These data imply that part of the SSc patients probably belong to the category of paraneoplastic syndrome with autoimmune manifestations.

### 2.2. Environmental Risk Factors and Their Modes of Action Associated with SSc

Until recently, a growing body of evidence has suggested that environmental factors play an initiating role in the alterations/modulations of epigenetic determinants for the onset and progression of genetically predisposed SSc. These environmental risk factors include silica, inorganic compounds, organic solvents, drugs, white spirits, vaccination [55,56,57,58,59,60], rapeseed oil [61], and heavy metals [62]. These environmental risks interact with genetic or epigenetic factors to breakdown the immune tolerance to self-antigens. Consequently, the autoantibodies are induced and tissue damage ensues in patients with SSc [62].

### 2.3. Aberrant Epigenetic Regulations in SSc

Epigenetics refers to reversible and stable hereditable modifications of gene expression and function but without alterations in DNA sequence [63]. The epigenetic regulation of gene expression includes DNA methylation, histone modification, and post-transcriptional mRNA regulation by non-coding RNAs [64]. DNA methylation is a biochemical process involving the transfer of a methyl group onto the C5 position of cytosine to form 5-methylcytosine at the position of a repeated CpG dinucleotides (CpG island) in the promoter region of a gene for repressing its expression [65]. The methylation of DNA is mediated by DNA methyltransferase (DNMT) 1, 3a, and 3b [66]. Conversely, gene transcription is achieved only after DNA demethylation which is activated by ten-eleven translocation (TET) enzymes, TET1, TET2, and TET3 [67]. On the other hand, post-transcriptional modifications of amino acid residues in histone may also alter chromatin structure. The enzymes involved in histone acetylation are histone acetyltransferases (HATs) and histone deacetylases (HDACs) which may regulate histone acetylation and up or downregulation of gene expression. Another two groups of enzymes involved in keeping histone methylation status, histone methyltranferases (HMTs) and histone demethylases (HDMs), may also down or upregulate the gene transcriptions. Besides, phosphorylation, ubiquitylation, and sumonylation can also modify histones for modulating DNA transcription [68,69,70].

**Figure 2:**
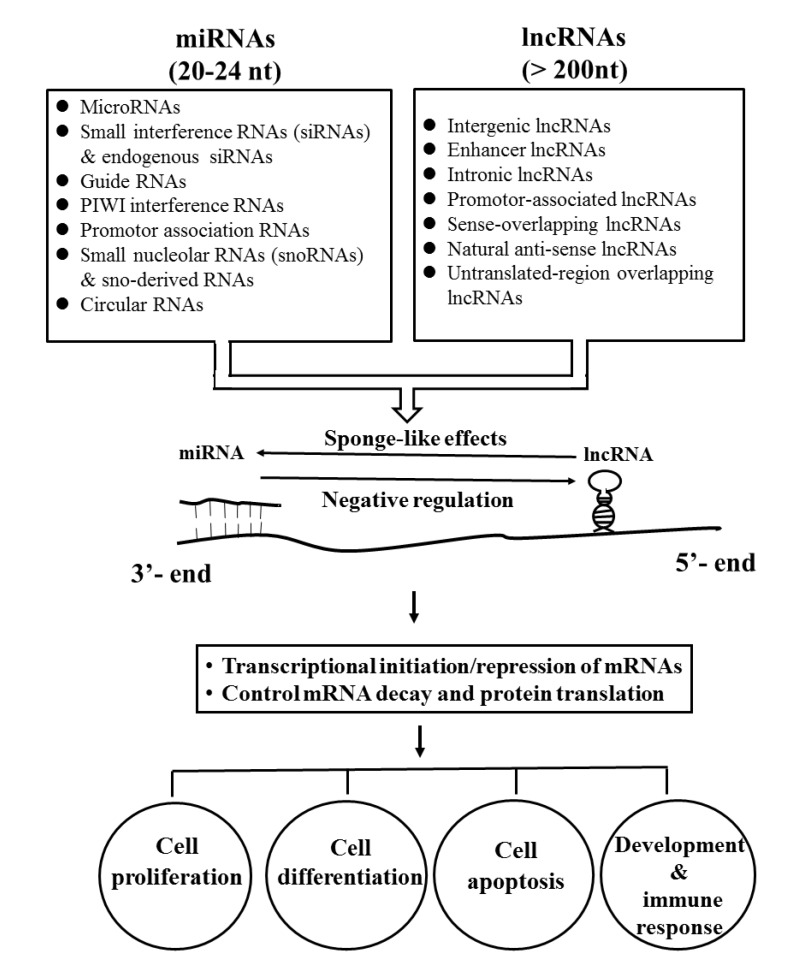
Different types of microRNAs (miRNAs) and long non-coding RNAs (lncRNAs) with their mutual interactions on the post-transcriptional regulation of mRNA expression and cell functions including proliferation, differentiation, apoptosis, and development of immune responses.

In addition to DNA methylation/demethylation and histone modifications, recent investigations have focused on the discovery of the regulatory functions of a group of “non-coding RNAs”, which cannot be translated to proteins. These RNA molecules have been categorized into two groups. The small molecules with nucleotide residues ranging between 20 and 24 nt are classified as microRNAs (miRs), whereas those with nucleotide residues more than 300 nt are classified as long non-coding RNAs (lncRNAs). miRs regulate gene expression by inducing transcription degradation or retarding RNA transferase activity through binding to a 3′-untranslated region (3′-UTR) of target mRNA, modulation of methylation in the DNA promoter regions, or modification of histone [71]. On the other hand, lncRNAs regulate gene expression by different mechanisms including epigenetic, transcriptional, post-transcriptional, translational, and peptide localization modifications [72,73,74,75]. Another unique feature of lncRNAs depends on their biochemical properties interacting with a wide range of molecules to form RNA-RNA, RNA-DNA, and RNA-protein complexes, indicating their vast functional diversities. Interestingly, interactions between lncRNAs and miRs have also been reported, i.e., lncRNAs can serve as sponge-like molecules to inhibit miR-mediated functions [76,77]. The functional classification of non-coding RNAs and their interactions for modulating mRNA expression and cell functions are illustrated in Figure 2. The different epigenetic modulations of immune-related cells in patients with SSc are discussed in detail in the following sections.

#### 2.3.1. Abnormal DNA Methylation in the Immune-Related Cells of Patients with SSc

Lei et al. [78] have measured the total methylation of CD4^+^ T cells in patients with SSc and found global hypomethylation due to decrease in DNMT1 and methyl-CpG-binding domain proteins (MBD), MBD3 and MBD4, together with their mRNAs expression in these immune cells. In addition, Wang et al. [79] and Almanzar et al. [80] have found DNA hyper-methylation in the FOXP3 promoter of CD4^+^T cells with decreased FOXP3 mRNA expression that subsequently led to Treg cell functional defect and hyperactivity of CCR6^+^Th cells. These results indicate that Th17/Treg imbalance predisposes inflammatory diathesis in patients with SSc. Zhu et al. [81] have unveiled that aberrant methylation regulation can potentially lead to differential expression of genes in peripheral blood mononuclear cells and be involved in the abnormal migration, proliferation, activation, and increased pro-inflammatory diathesis of immune-related cells in patients with SSc.

#### 2.3.2. Abnormal Histone Modifications in the Immune-Related Cells of Patients with SSc

Wang et al. [82] have reported the occurrence of global histone H4 hyperacetylation as well as global histone hypomethylation in the B cells of SSc patients. Moreover, they noted that the global histone H4 acetylation and HDAC2 expression were negatively correlated whereas global histone H3K9 methylation was positively correlated with SUV39 H2 protein expression. They concluded that the altered histone modifications in B cells of SSc patients were associated with skin thickness as well as increased disease activity in SSc. It is thus inferred that a number of autoantibodies produced by hyperactive B cells can cause different clinical manifestations and pathological changes including vasculopathy and tissue fibrosis in patients with SSc, which originate from abnormal histone modifications (Table 1). Ciechomska et al. [83] have further demonstrated that histone demethylation in conjunction with Toll-like receptor (TLR)-8 activation in monocytes could promote trans-differentiation of FBs to MFBs via an activator protein 1 family member, Fra-2. The increased trans-differentiation from FBs to MFBs inevitably enhances the extracellular matrix synthesis and accumulation, and finally tissue fibrosis. Current investigations have also suggested that the proliferation-boosting cytokines may trigger epigenetic changes and persistently activate the phenotypic trans-differentiation of FBs [84].

#### 2.3.3. Enhanced DNA Hypomethylation in the Dermal Fibroblasts of SSc Patients

Hattori et al. [85] have found the expression level of TET1 mRNA in SSc-dermal FBs is 1.68-fold higher than in normal dermal FBs. The expression levels of DNMT1 and DNMT3B mRNA also show an increased tendency in SSc-FBs. Moreover, the TET1 expression in these dermal FBs is abnormally regulated in hypoxic environment and accompanied by a global DNA hypomethylation. Altorok et al. [86] conducted a genome-wide DNA methylation study of dermal FBs obtained from SSc patients. They found only 203 CpG loci in 485,000 methylation sites across the whole genome were differentially methylated in both diffuse and localized SSc patients. The common hypomethylated genes include *ITGA*9 (encoding an α integrin), *ADAM12*, *COL23A1*, *COL4A2,* and *MYO1E*, together with their transcriptional factor genes, *RUNX1*, *RUNX2*, and *RUNX3*. Further analyses unfolded that the genes involved in the extracellular matrix–receptor interaction and focal adhesion were all enriched in the dermal FBs of patients with SSc. These data may suggest a concept that SSc patients with a genetic predisposition in their FBs can spontaneously exhibit abnormal epigenetic regulation in their collagen fiber producing cells.

In short conclusion, aberrant genetic and epigenetic regulations in patients with SSc facilitate the immune-related cells and dermal FBs to move toward a fibrinogenetic diathesis after stimulations by environmental factors.

## 3. Cellular and Molecular Mechanisms for Tissue Fibrosis in Patients with SSc

### 3.1. Pathophysiology of Myofibroblasts and Other Connective Tissue Cell Lineages in Patients with SSc

**Figure 3:**
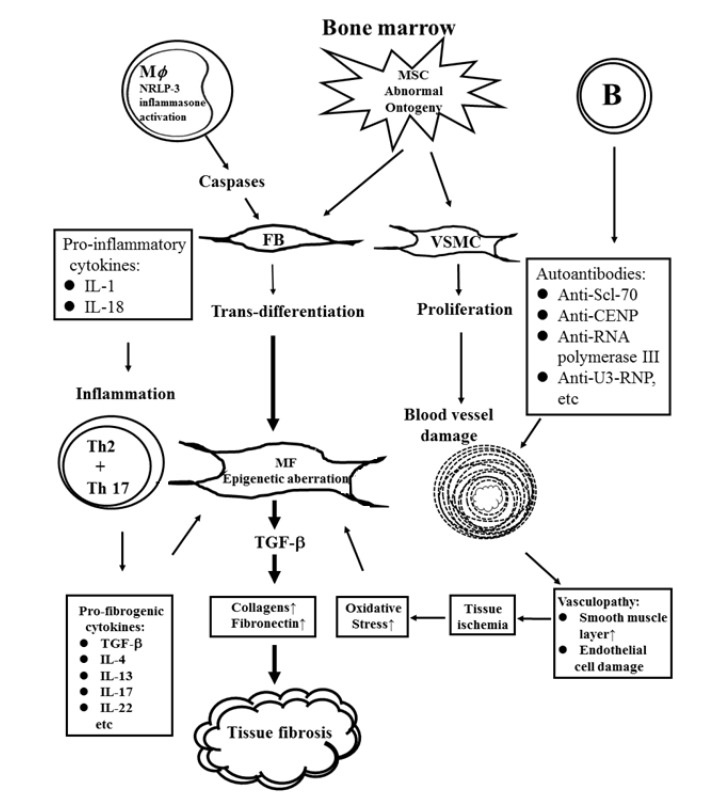
The underlying cellular and molecular bases of vasculopathy, chronic inflammation, and tissue fibrosis in patients with systemic sclerosis via pro-inflammatory cytokines, pro-fibrogenic cytokines, autoantibodies, caspases, and oxidative stresses to elicit trans-differentiation of fibroblasts to myofibroblasts and finally tissue fibrosis in the patients. M*φ*: macrophage, MSC: bone marrow-derived mesenchymal stem cell, FB: fibroblast, MFB: myofibroblast, VSMC: vascular smooth muscle cell.

Tissue fibrosis is the most lethal condition in patients with SSc. Persistent activation of MFBs is responsible for the overproduction and accumulation of extracellular matrix and fibronectin in different tissues and organs of the patients [87,88]. Many investigators have also found that a variety of connective tissue cell lineages including resident FBs/fibrocytes, keratinocytes, endothelial cells, pericytes, pre-adipocytes/adipocytes, and resident tissue stromal cells are implicated in the fibrosis of SSc patients [89,90]. Many different immune-related cells such as M2 macrophages [91,92], dendritic cells (DCs) [92,93], mast cells [94], neutrophils [95], B lymphocytes [96,97], T lymphocytes [98,99], innate lymphoid cells [100], endothelial cells [101], platelets [14,102], adipocytes [103], and keratinocytes [104] are involved in the modulation of tissue fibrosis in SSc. The caspases released from the activated NLRP-3 inflammasomes of innate immune cells can facilitate FB-MFB trans-differentiation. In addition, the inflammatory cytokines such as IL-1 and IL-18 released from activated macrophages stimulate tissue inflammation and MFB trans-differentiation, and further increase collagen fiber syntheses. The skewed Th2 and Th17 populations produce pro-fibrotic cytokines (TGF-β, IL-4, IL-13, IL-17, IL-22, etc.), which further stimulate MFBs and tissue inflammation. These events subsequently facilitate extracellular matrix synthesis and deposition. The autoantibodies produced by B cells may destroy vascular endothelial cells and enhance vascular smooth muscle hypertrophy as well as ultimate vasculopathy. These intriguing interactions among immune-related cells, cytokines, and connective tissue cells are shown in Figure 3.

#### 3.1.1. Aberrant Ontogenesis of Mesenchymal Stem Cells (MSCs) and Abnormal Cellular Physiology of Their Descendant Vascular Smooth Muscle and Endothelial Cells in Patients with SSc

Di Benedetto et al. [105] assessed the miR expression profiles of the bone marrow-derived MSCs (BM-MSCs) and adipose tissue-derived MSCs (A-MSCs) from patients with SSc. They have found that both lineages from SSc patients express extraordinarily high levels of miRs associated with senescence and pro-fibrotic tendency. Their results have suggested the pro-fibrotic properties of stem cells in SSc. Hegner et al. [106] further demonstrated that disturbed endogenous regeneration capacity in SSc-MSCs skewed vascular smooth muscle cell (VSMC) differentiation toward MFB lineage. Moreover, Mendoza et al. [101] have reported that the CD31^+^/CD102^+^ endothelial cells obtained from lung tissues of SSc patients with interstitial lung disease (ILD) expressed high levels of MSC specific genes (COL-I, COL-III, and fibronectin), EC-specific genes (COL-IV and vascular endothelial cadherin), pro-fibrotic genes (TGF-β and CTGF), and genes encoding transcription factors for the transition from endothelial-to-mesenchymal cells (EndoMT), as well as the related transcription factors, TWIST1 and SNAI2. EndoMT refers to a trans-differentiation by which ECs lose their specific morphology/markers to acquire MFB-like properties. Following this line of discovery, Manetti et al. [107] reported that dermal microvascular EC obtained from SSc patients (SSc-dMVECs) exhibited not only a spindle-shaped appearance but also an existence of low levels of CD31 and VE-cadherin with abundant MFB markers (α-SMA^+^ stress fibers, S100A4, and type 1 collagen). They concluded that EndoMT in SSc may potentially become a crucial process linking endothelial dysfunction and development of dermal fibrosis. Furthermore, Zhao et al. [108] demonstrated that overexpression of lncRNA, HIFα-anti-sense RNA1 (HIFα-AS1), enhanced the expression of caspase 3, caspase 8, and Bcl-2 in VSMC of SSc patients. These factors increased proliferation and decreased apoptosis of VSMCs in SSc patients complicated with thoraco-abdominal aortic aneurysm. The detailed dissection of the cellular and molecular mechanisms underlying the pathogenesis of tissue fibrosis in patients with SSc is illustrated in Figure 1 and Figure 3.

#### 3.1.2. Histological Characteristics and the Biochemical Constituents in Tissue Fibrosis of Patients with SSc

The specific histological findings and biochemical constituents of SSc-skin are the deposition of collagen (COL) I, III, and V, which are co-assembled into a unique macromolecule to form heterotypical fibers [109,110]. COL-V is the minor component bridging between COL-I and COL-III where it contributes to the development of functional connective tissues [111,112]. Parra et al. [113,114] and Martin et al. [115] have observed an increased COL-V expression in SSc-lung associated with reduced vital capacity and diffusion capacity for carbon monoxide. In addition to collagens, fibronectin expression is also enhanced by oxidative stress-activated FBs. The anti-oxidant such as epigallocatechin-3-gallate can modulate COL I, fibronectin, and dermal FB activity in SSc as reported by Dooley et al. [116]. Imbalance between matrix metalloproteinases (MMPs) and tissue inhibitor of metalloproteinase (TIMP) might also contribute to excessive accumulation of collagen fibers in SSc-dermis as reported by Verrechia et al. [117].

### 3.2. Tissue Fibrosis-Related Cytokines and Their Signaling Pathways in SSc

A number of autoimmune-related cytokines and growth factors are supposed to be implicated in the tissue fibrosis in SSc patients. Among which, transforming growth factor-β (TGF-β) is regarded as the master regulator in the development of tissue fibrosis in these patients [117,118,119,120]. The increased expression of TGF-β by Th2 cells, macrophages, fibroblasts, myofibroblasts, and keratinocytes can enhance a synthesis and deposition of extracellular matrix in situ via both canonical (Smad 2/3) and non-canonical (Smad 4) adaptor proteins of Wnt/β-catenin signaling pathways. Despite the profibrosis-inducing effect of TGF-β, the cytokine may also inhibit transcription factor, GATA-3, to suppress IL-13 and IL-5 expression from Th2 cells, acting as an anti-inflammation process in a negative feedback loop. Tang et al. [119] and Hu et al. [120] have reported that TGF-β can enhance Smad 2 and Smad 3, but suppress Smad 7 in MFBs. Recent studies have further revealed that TGF-β can exert an additional effect on the regulation of TGF-β1-Smad signaling pathway via ncRNA modulations as well as epigenetic modifications of DNA and histones [118,119]. Meng et al. [118] further demonstrated that both Wnt/β-catenin signaling pathway and lipid metabolism were concomitantly transduced by TGF-β1. In addition, Trojanowska M [121] reported that cross-talk between TGF-β and platelet derived growth factor (PDGF) signaling pathways could regulate chronic tissue fibrosis in SSc. In addition to TGF-β, Artlett et al. [122] have reported that IL-1 cytokine family including IL-1α, IL-1β, IL-18, IL-33, IL-36α, IL-36β, IL-36γ, and IL-38 can also contribute to the skin inflammation and fibrosis in patients with SSc. Kotsiou et al. [123] and Xu et al. [124] have further demonstrated that the IL-33/ST2 axis signaling pathway is involved in SSc and other fibrotic diseases. O’Reilly et al. [125] and Nquyen et al. [126] reported that Th2 polarization by overexpression of IL-4/IL-13 axis contributes to the initiation and perpetuation of collagen deposition in fibrotic skin and scarring diseases of SSc patients via STAT6 signaling and miR-135b modulation. Wang et al. [127] found that tissue hypoxia accelerated multifunctional reprogram of FBs to produce IL-6 via an upregulation of the TGF-β1 signaling pathway. Robak et al. [128] found that higher levels of IL-17B and IL-17E were associated with both localized and diffuse SSc whereas high IL-17F was associated only with localized SSc. On the contrary, Nakashima et al. [129] have demonstrated that IL-17A exerts an anti-fibrotic effect via upregulation of miR-129-5p and downregulation of connective tissue growth factor (CTGF) and α1 collagen. In SSc FBs, IL-17A signaling is suppressed due to downregulation of its receptor by the intrinsic TGF-β1 activation. To further explore the IL-17 downstream signaling pathway in SSc, Ahmed et al. [130] have demonstrated that PD-1, SHP2, STAT3, Ras/Erk, mTOR and complement components are all involved in this particular signaling pathway. Another important cytokine associated with SSc fibrosis is IL-22. It is a member of the IL-10 cytokine family mainly produced by CD4^+^T cells and innate lymphoid cells. Sawamura et al. [131] have demonstrated that IL-22 expression in SSc skin infiltrated with lymphocytes, can potently upregulate COL I production by dermal FB via let-7a downregulation. On the contrary, IL-35, a heterodimeric cytokine belonging to the IL-12 family, exhibits an anti-fibrotic activity and has been found suppressed in SSc by Luo et al. [132]. 

Besides the above-mentioned cytokines, certain growth factors, miRs, and biomolecules have been found able to modulate tissue fibrosis in SSc patients. These may include endoglin, a co-receptor for TFG-β reported by Maring et al. [133], extracellular secreted protein acidic and rich in cysteine (SPARC) reported by Carvallieiro et al. [134], and intracellular E_3_ ubiquitin ligase reported by Huang et al. [135]. All of the three molecules can stimulate the pro-fibrotic activity of dermal FBs obtained from SSc. In contrast, sirtuins (SIRTs), a group of histone deacetylases with anti-fibrotic activity, are decreased in SSc as demonstrated by Wyman et al. [136].

Table 2 summarizes the fibrosis-related cytokines, growth factors, and molecules, and their major signaling/modes of action in patients with SSc.

### 3.3. Tissue Fibrosis-Related ncRNAs and Their Signaling Pathways in Patients with SSc

#### 3.3.1. Tissue Fibrosis-Related miRs in SSc

**Figure 4:**
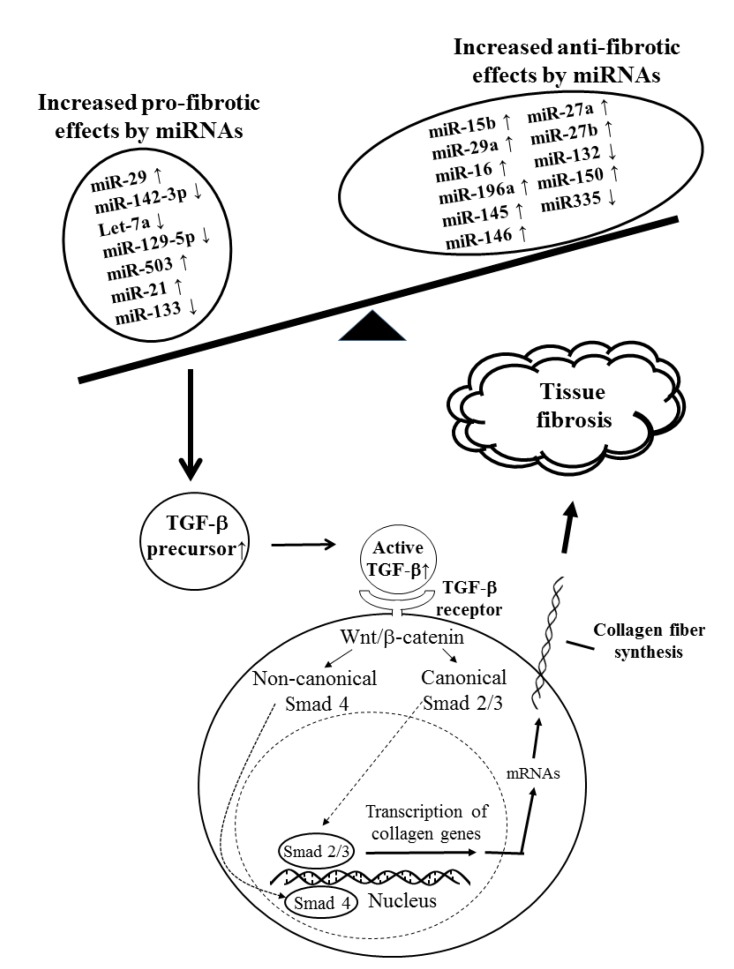
Imbalance between pro-fibrotic- and anti-fibrotic-related microRNAs skews the naïve helper T cell toward Th2 subpopulation. The TGF-β secreted from Th2 cell binds to TGF-β receptors on myofibroblasts. The binding transduces signals via both canonical (Smad 2/3) and non-canonical (Smad 4) Wnt/β-catenin pathways to transcribe the expression of collagen genes, *COL-I, COL-II*, and *COL-IV*. Finally, excessive collagen fiber synthesis and tissue fibrosis ensue. **↑**: upregulation, **↓**: downregulation.

It is conceivable that approximately 50% miRs are expressed from non-protein coding transcripts. The rest are located in the introns of the coding genes and are co-transcribed with their host genes, but are separately processed for intracellular modulation of gene expression. Besides, miRs are also contained in the extracellular small vesicles such as exosomes. Exosomes are micro-vesicles encapsulated by lipid bilayer, containing insides various biomolecules such as proteins, lipoproteins, carbohydrates, DNAs, mRNAs and miRs, to act as vehicles for inter-cellular or inter-tissue communications. Previous investigators have demonstrated that many miRs are involved in the fibrinogenesis in SSc. These fibrosis-related miRs can be classified into pro-fibrotic and anti-fibrotic miRs detected in the dermal FBs [137,138] or circulating exosomes [139,140] in patients with SSc. Once the imbalance between pro-fibrotic and anti-fibrotic miRs in Th2 cells of SSc patients occurs toward profibrotic end, some mechanisms would trigger TGF precursors toward active form of TGF-β in Th2 cells. The released active form of TGF-β then binds to TGF-β receptors on MFBs, initiating a transcription and synthesis of collagen fibers through Wnt/β-catenin signaling pathways [49,141,142,143,144,145] as mentioned in the above (Section 3.2). Figure 4 illustrates the imbalance between pro- and anti-fibrotic miRs with a skewing toward fibrinogenesis, which transduces fibrosis signaling in patients with SSc.

#### 3.3.2. Tissue Fibrosis Relevant Long Non-Coding RNA in SSc

Thanks to the development of next generation sequencing (NGS) technology, particularly RNA sequencing, various investigations have revealed that there are 92,343 lncRNA genes in a whole human genome, which is twice as many as the human protein coding genes [146]. These large numbers of lncRNA can be functionally classified into seven groups including intergenic, enhancer, promoter-associated, sense-overlapping, natural anti-sense, intronic, and untranslated-region overlapping lncRNAs [147]. These functional lncRNAs can serve as important epigenetic regulatory factors for gene expression, genetic imprinting, histone modifications, chromatin dynamics, and interactions with other molecules such as miRs and proteins in the somatic and immune-related cells [73,148,149,150]. Aberrant expression of lncRNAs has been explored in many autoimmune diseases such as systemic lupus erythematosus, rheumatoid arthritis, type I diabetes, autoimmune thyroid diseases, multiple sclerosis, polymyositis/dermatomyositis, psoriasis, and Crohn’s disease in the literature [43,151]. As investigated in immunology/rheumatology realm, overactive Th17 cells play important roles in the pathogenesis of many autoimmune/inflammatory diseases. Teimuri et al. [152] have identified the expression of lncRNAs, AL450992.2, AC009948.5, and RP11-98D18.3, as Th17 cell-lineage specific lncRNAs and their levels can serve as new potential biomarkers in autoimmune and inflammatory diseases. 

In contrast to many studies on miRs-related tissue fibrosis in patients with SSc as shown in Section 3.3.1 and Figure 4, the reports on the lncRNA-associated tissue fibrosis in SSc are relatively rare in the literature. Wang et al. [153] have discovered that increased serum level of TSIX represents an lncRNA biomarker with stabilization activity on collagen mRNA. The upregulation of TSIX seen in dermal FBs of SSc patients may originate from activation of endogenous TGF-β signaling with eventual enhancement of collagen synthesis by these cells. Mariotti et al. [154] have demonstrated that lncRNA NRIR (a negative regulator of interferon response), is significantly upregulated in monocytes of SSc patients. This may account for the increased interferon (IFN) signature, autoimmunity, and auto-inflammatory nature of patients with SSc. Recently, Dolcino et al. [155] have identified a unique lncRNA 00201 that regulates genes involved in four main features of SSc, i.e., fibrosis, vasculopathy, autoimmunity, and carcinogenesis. Messemaker and colleagues [156], by using skin biopsy-derived RNAs from SSc patients, have discovered a number of elevated anti-sense lncRNA expression in SSc skin. They identified three dysregulated lncRNAs, CTBP1-AS2, OTUD6B-AS1, and AGPP2-AS1, which were relevant to skin fibrosis. Takata et al. [157] further confirmed a downregulation of OTUD6B-AS1 expression in SSc-FBs and human pulmonary artery smooth muscle cells (HPASMC) after PDGF stimulation. Silencing of this particular lncRNA could significantly enhance cyclin D expression. Knockdown of OTUD6B-AS1 significantly reduced proliferation of and suppressed apoptosis of both dermal FBs and HPASMC. These results have suggested that OTUD6B-AS1 regulates cell proliferation and apoptosis via cyclin D1 expression in a sense gene-independent manner and the apoptosis-resistance mechanism in FBs and vascular smooth muscle cells is relevant to OTUD6B-AS1 function and the development of tissue fibrosis in patients with SSc.

The lncRNAs-related tissue fibrosis, hypertrophy of vascular smooth muscle cells, autoimmunity/inflammation, and their respective target mRNAs in patients with SSc are listed in Table 3. However, the downstream signaling pathways and relationships to the fibrosis-related cytokines need further investigation.

**Figure 5:**
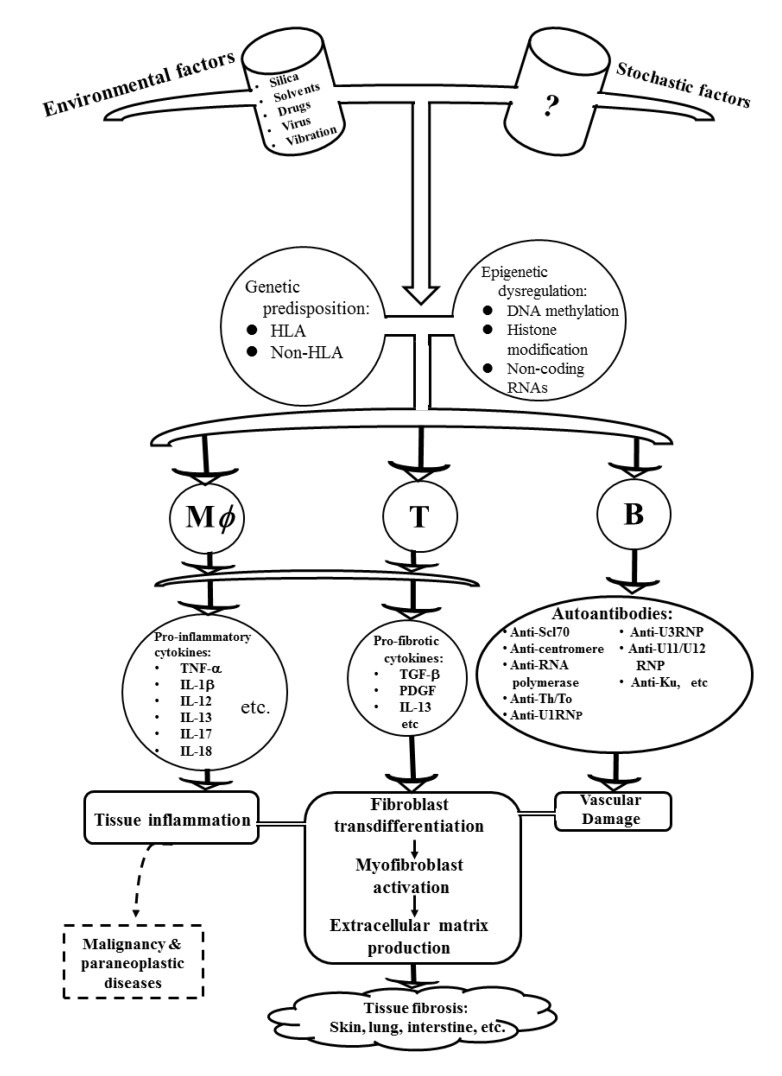
A scheme depicting in detail the contributing factors including genetics/epigenetics predispositions, environmental factors, and the undefined stochastic stresses in inducing aberrant immune responses. These factors may cause vascular endothelial cell damage, vascular smooth muscle hypertrophy, chronic inflammation, autoantibody productions, excessive oxidative stress, and aberrant non-coding RNA expression. These pathological modalities lead to increased trans-differentiation of fibroblasts to myofibroblasts. Finally, tissue fibrosis occurs in the patients with systemic sclerosis.

Putting all of the SSc-related pathogenic factors together, a scheme depicting the potential contributing factors for tissue fibrosis and paraneoplastic syndrome is shown in Figure 5.

## 4. Potential Biomarkers and New Therapeutic Strategy for Patients with SSc

In addition to the intracellular ncRNA regulation in mRNA expression, extracellular vesicles (EV) released from all cell types also play a role in the intercellular communications such as regulation of chronic inflammation or immune responses [158]. Transcriptional and proteomic analyses of EVs purified from patients with SSc can be expected to become the useful tools for finding biomarkers to help diagnosis, classification, assessment of disease activity, providing prognosis and evaluation of therapeutic effectiveness in patients with SSc [143]. Stypinska et al. [159] have reported miR expression profiles in cell-free serum from patients with different autoimmune diseases including SLE, RA, mixed connective disease (MCTD), and SSc. Moreover, Chouri et al. [160] have demonstrated serum miR-483-5p as a potential driver of fibrosis in SSc, and Rusek et al. [161] have reported an upregulation of novel serum miR-4484 associated with increased MMP-21 expression in SSc. This evidence may indicate some unique circulatory miRs are not only useful biomarkers but pathogenic indicators for SSc. Up to the present, there is no report regarding the expression profile of exosomal lncRNAs in the serum of SSc patients. It is expected that certain exosomal miRs and lncRNAs identified in serum will become useful biomarkers for pathological manifestations in SSc patients in the future.

In the cellular level, Manetti et al. [13] have demonstrated circulatory T_ang_ cell expansion in SSc patients with severe peripheral vascular damage. This may imply that the level of peripheral blood T_ang_ population can become a potential cellular biomarker in these patients.

Since the revealing of epigenetic regulations of gene expression including DNA methylation/acetylation, histone modifications, and ncRNA in SSc pathogenesis, many authors have reported that the epigenetic modifications can also be achieved by chemical and epigenetic editing technology. Wang et al. [162] and Hemmantazad et al. [163] have reported that trichostatin A, an HDAC inhibitor, could silence HDAC-7 of SSc-FBs. Chan et al. [164] have found that 5-aza-2′- deoxycytidine, a DNMT inhibitor, could induce hypomethylation of FL11, DKK1, and SFRP1 in SSc-FBs. Dees et al. [165] have demonstrated that azacytidine could inhibit the Wnt pathway by targeting DNMT1 in SSc-FBs.

An miR targeting strategy has also been designed by using miR-targeting anti-sense oligonucleotide (anti-miRs), which are highly complementary to the target miR [166]. These anti-miRs could suppress target miR function, blocking their inhibitory effect on the expression of endogenous target genes after delivery by lentiviral vector. Furthermore, miR-masking technologies (miR-mask) have become another strategy for anti-sense oligonucleotide approaches [167]. On the other hand, over the past years, a cutting-edge epigenetic engineering called epigenetic editing, by use of CRISPR/Cas9 system, has been developed. This tool, acting as a highly efficient site-specific DNA binding domain, would become a novel epigenetic editing module for inhibiting aberrant ncRNAs regulation [168,169].

Wang et al. [149] and O’Reilly et al. [170] have reported that silencing lncRNA, TSIX, can result in a reduction in COL 1 level in SSc FBs. In addition, Li et al. [171] have demonstrated that lncRNA, CIR, can promote extracellular matrix degradation in chondrocytes of patients with osteoarthritis by acting as a sponge for miR-276. Speculatively, it is also possible that a modulation of TGF-β signaling pathway by sense or anti-sense lncRNAs can control the pro-fibrotic processes in SSc-FBs and may serve as a new therapeutic strategy for treating SSc fibrosis in the future.

## 5. Conclusions and Prospects

SSc is a systemic autoimmune disease constellated with multi-organ fibrosis, vasculopathy, and autoimmunity, which are characterized by the presence of pro-inflammatory/pro-fibrotic/anti-fibrotic cytokines, autoantibodies, and carcinogenesis. Overexpression of β-catenin signaling pathways play a master regulatory role in the tissue fibrosis of patients with SSc. Evidence supports that TGF-β and its downstream signaling are regulated by genetic, epigenetic, and environmental factors. A number of miRs have been found closely related to tissue fibrosis in patients with SSc. However, only a few lncRNAs have been reported relevant to tissue fibrosis in patients with SSc. It is expected that some epigenetic regulatory molecules, particularly the serum exosomal fibrosis-related lncRNAs, may not only become useful biomarkers for monitoring, diagnosing, and predicting prognosis, but serve as potential therapeutic targets in SSc patients in the future.

To accomplish the aforementioned unmet needs, we propose the following issues may become future foci for investigations: (1) identification of more specific fibrosis-related lncRNAs in SSc-FBs; (2) development of more specific biochemical routes for epigenetic modification of SSc-FBs; (3) development of ncRNA modulators such as miR-mask or anti-miR to aim against pathogenic pre-miRs in SSc-FBs; and (4) development of new epigenetic editing technologies i.e., CRISPR/Cas9 system, for the future intervention in patients with SSc.

## Figures and Tables

**Table 1:** Autoantibody-related clinical manifestations in patients with systemic sclerosis (SSc).

Autoantibody	Clinical Manifestation	References
Anti-topoisomerase 1 (anti-Scl-70)	Pulmonary fibrosis Cardiac involvement Malignancy Raynaud’s phenomenon	[16,17,18]
Anti-centromere proteins B and C	Raynaud’s phenomenon Ischemic digital loss Sicca syndrome	[18,19,20]
Anti-RNA polymerase III	Skin fibrosis Renal crisis	[21,22]
Anti-U_3_-RNP (fibrillarin)	Pulmonary arterial hypertension Cardiac involvement Skeletal muscle involvement	[23,24,25,26]
Anti-U_11_/U_12_-RNP	Pulmonary fibrosis	[27,28]
Anti-B_23_	Pulmonary hypertension Lung diseases	[29,30]
Anti-Ku	Muscle and joint involvement	[31,32]
Anti-Th/To-RNP	Lung diseases Renal crisis Small-bowel involvement	[33,34]
Anti-endothelial cells	Skin and lung fibrosis	[35]
Anti-fibroblast	Skin and lung fibrosis	[36]
Anti-metalloproteinase 1	Extracellular matrix deposition	[37]
Anti-M_3_-muscarinic receptor	Gastrointestinal dysmotility Sicca	[38]
Anti-PDGFR	Tissue fibrosis	[39]
Anti-cardiolipin/phospholipid	Vasculopathy	[40]
Anti-ICAM-1	Endothelial dysfunction	[41]
Anti-fibrillin-1	Tissue fibrosis	[42,43]

**Table 2:** The fibrosis-related cytokines/growth factors/molecules, and their signaling/modes of action in patients with systemic sclerosis.

Fibrosis-Related Cytokines/Molecules	Signaling/Modes of Action
**[I] Pro-fibrogenic cytokines:**	
TGF-β [117,118,119,120]	Smad 2/3, Wnt/β-catenin
PDGF [121]	
IL-1 family (IL-1, IL-33, IL-36) [122,123,124]	
IL-4/IL-13 [125,126]	STAT6, miR-135b
IL-6 [127]	
IL-17B, IL-17E, IL-17F [128,129,130]	
IL-18 [122]	
IL-22 [131]	let-7a ↓→ collagen I ↑
IL-33 [122]	ST2 (suppressor of tumorigenicity 2 receptor)
**[II] Fibrosis-related molecules:**	
Endoglin (co-receptor for TGF-β signaling) [133]	Smad 2/3, Wnt/β-catenin
SPARC (secreted protein acidic and rich in cysteine) [134]	Smad 2/3, Wnt/β-catenin
E3 ubiquitin ligase [135]	Ubiquitin-mediated degradation of TGF-β/Smad signaling pathway

**[III] Anti-fibrinogenic cytokines:**	
IL-17A [129]	miR-129-5p↑→CTGF *↓
IL-35 [132]	
**[IV] Anti-fibrogenic molecules:**	
Sirtuins (histone deacetylase) [126]	TGF-β inducing signaling↓
	mTOR signaling↓
	Oxidative stress↓
	Cell senescence marker p-21↓

* CTGF: connective tissue growth factor; **↑**: upregulation; **↓**: downregulation.

**Table 3:** Involvement of aberrant lncRNA expression, the target mRNA, and pathological changes in patients with systemic sclerosis.

lncRNA	Expression Level	Tissue or Cell Type	Target mRNA	Pathology
TSIX [149]	↑	Dermal fibroblast, skin tissue and serum	Type I collagen mRNA stabilization	Fibrosis
NRIR [150]	↓	Peripheral blood monocytes	Type 1 IFN and its stimulated mRNA	Autoimmuity, Inflammation
ncRNA00201 [151]	↓	Peripheral blood mononuclear cells	EGFR Enb B1 S1P1 ALK1 Endothelins RhoA MAPK Class I-PI3K mTOR TGF-βR MyD88 TLRs RAC	Autoimmunity, Vasculopathy, Fibrosis, & Carcinogenesis
OTUD6B-AS1 [49,152,153]	↓	Skin tissue, Fibroblast *, HPASMC	Cyclin D1	Fibrosis, Vasculopathy
CTBP1-AS2 [49,152]	↑	Skin tissue	ND	ND
AGAP2-AS1 [49]	↑	Skin tissue	ND	ND
HIFA-AS1 [108]	↑	Vascular smooth muscle cells	Bcl-2 Caspase 3 and 8	Vasculopathy

* HPASMC: human pulmonary arterial smooth muscle cell; **↑**: upregulation; **↓**: downregulation.

## Abbreviations

anti-CENP	anti-centromere protein antibody
anti-ICAM-1	anti-intercellular adhesion molecule-1 antibody
anti-PDGFR	anti-platelet derived growth factor receptor antibody
anti-TOPO-1	anti-topoisomerase-1 antibody (anti-Scl-70)
A-MSC	adipocyte derived mesenchymal stem cell
AS	anti-sense non-coding RNA
BM-MSC	bone marrow derived mesenchymal stem cell
CIR	cartilage injury related long non-coding RNA
COL	collagen
CTGF	connective tissue growth factor
DC	dendritic cell
EC	endothelial cell
ECM	extracellular matrix
EndoMT	trans-differentiation from endothelial cell to mesenchymal cell
EV	extracellular vesicle
FB	fibroblast
HIF	hypoxia-induced factors
HPASMC	human pulmonary arterial smooth muscle cell
IL	interleukin
ILD	interstitial lung disease
lncRNA	long non-coding RNA
MFB	myofibroblast
miR	microRNA
MMP	matrix metalloproteinase
mRNA	messenger RNA
MSC	mesenchymal stem cell
NLRP-3	neuronal apoptosis inhibitor protein, leucine-rich repeat, pyrin domain containing protein 3
NRIR	a negative regulator of interferon response
PDGF	platelet derived growth factor
S100A4	S100 calcium-binding protein A4
SNAI2	Snail superfamily of C_2_H_2_-type zinc finger transcription factor 2
SPARC	secreted protein and rich in cysteine
SSc	systemic sclerosis
SSc-dMVEC	dermal microvascular endothelial cell obtained from systemic sclerosis
T_ang_	angiogenic T cell
TIMP	tissue inhibitor of metalloproteinase
TGF-β	transforming growth factor-β
Th	helper T cell
TWIST1	Twist related protein 1 or class A basic helix–loop–helix protein 38 (bHLHa38)
VE	vascular endothelium
VSMC	vascular smooth muscle cell
